# An Interim Analysis of a Randomized, Open-Label Study of Vilazodone, Escitalopram, or Vortioxetine for Major Depressive Disorder

**DOI:** 10.7759/cureus.37858

**Published:** 2023-04-20

**Authors:** N Simple Santi, Sashi Bhusan Biswal, Birendra Narayan Naik, Jyoti Prakash Sahoo, Bhabagrahi Rath

**Affiliations:** 1 Pharmacology, Veer Surendra Sai Institute of Medical Sciences and Research, Burla, IND; 2 Psychiatry, Veer Surendra Sai Institute of Medical Sciences and Research, Burla, IND; 3 Pharmacology, Kalinga Institute of Medical Science, Bhubaneswar, IND

**Keywords:** escitalopram, vilazodone, vortioxetine, selective serotonin reuptake inhibitor, montgomery-asberg depression rating scale, hamilton depression rating scale, depressive disorder

## Abstract

Introduction: The troubling issues of conventional antidepressants are inadequate disease remission and potential adverse effects. There is a dearth of research findings comparing vilazodone, escitalopram, and vortioxetine. The objective of this analysis is to determinechanges in the Hamilton Depression Rating Scale (HDRS) and Montgomery-Åsberg Depression Rating Scale (MADRS) scoresand the incidence of adverse events at 12 weeks.

Methods: This is an exploratory interim analysis of a randomized, three-arm, open-label ongoing study. The participants were randomly assigned in a 1:1:1 ratio to receive either vilazodone (20-40 mg/d), escitalopram (10-20 mg/d), or vortioxetine (5-20 mg/d). Efficacy and safety assessments were done at baseline, four weeks, eight weeks, and 12 weeks.

Results: Forty-nine(69%) of the 71 enrolled participants (mean age 43.9±12.2 years; 37 men (52%)) completed the 12-week follow-up. At baseline, the three groups' median HDRS scores were 30.0, 29.5, and 29.0 (p=0.76), respectively, and at 12 weeks, they amounted to 19.5, 19.5, and 18.0 (p=0.18), respectively. At baseline, group-wise median MADRS scores were 36, 36, and 36, respectively (p=0.79); at 12 weeks, they were 24, 24, and 23, respectively (p=0.03). In the post-hoc analysis, the inter-group comparison of the change in HDRS (p = 0.02) and MADRS (p = 0.06) scores from baseline did not reach statistical significance. No participants experienced serious adverse events.

Conclusion: In this initial assessment of a continuing study, vortioxetine exhibited a clinically (not statistically) significant drop in HDRS and MADRS scores, compared to vilazodone and escitalopram. The antidepressant effects need to be investigated further.

## Introduction

The past two decades have witnessed an unprecedented increase in the global burden of depressive disorder [1]. Far and wide, an astounding 322 million people have battled depression at some stage [2]. The World Health Organization (WHO) issued a warning that depression is supposed to surpass ischemic cardiovascular disorders as the leading global cause of morbidity in the years ahead [3]. One in 20 Indians, according to the country's National Mental Health Survey 2015-16, experienced depression [4]. In line with the latest statistics, India's annual prevalence of major depressive disorder (MDD) is 15.9% [5]. MDD was the leading driver (26.7%) of disability-adjusted life years (DALYs) in India in 2017 [6]. The physical, psychological, social, and financial facets of health are seriously jeopardized by MDD [7-9].

While there is an array of antidepressants at hand, it is still unclear which drug is most advantageous for MDD. It can be traced to unsustainable remission and the lackluster safety profile of today's drugs [10]. Consequently, there is debate over whether newer medications should take the forefront in managing MDD instead of existing antidepressants. Escitalopram, the S-enantiomer of citalopram, is one of the most widely prescribed selective serotonin reuptake inhibitors (SSRIs) for controlling MDD. It has also showcased its modulatory effects at an allosteric binding site of the serotonin transporter [11]. Through its selective inhibition of 5-HT reuptake and partial agonistic activity at 5-HT1A receptors, vilazodone strikes both monoamine transporters and receptors. Therefore, it is anticipated to be effective and well-tolerated in MDD patients [12]. A serotonin modulator, vortioxetine, also inhibits the serotonin transporter and directly modulates serotonin receptor function. In depressed patients who have not responded well to SSRIs or serotonin and norepinephrine reuptake inhibitors (SNRIs), it has demonstrated promising effects as an alternative remedy [13]. This study was bolstered by speculation that antidepressants with unique pharmacological mechanisms might provide an appealing solution for patients with MDD. The patients' scrupulous adherence to the treatment they received was the most fundamental challenge to the study's feasibility. Recent scientific studies [14-19] and meta-analyses [20,21] support the above drugs from their perspectives.

This study aimed to determine the efficacy and safety of antidepressant monotherapy following a 12-week course of interventions with vilazodone, escitalopram, or vortioxetine. Here we chronicle the interim results focused on changes in the Hamilton Depression Rating Scale (HDRS)-17 items version [22] and Montgomery Åsberg Depression Rating Scale (MADRS) [23] scores as well as the incidence of adverse events of a more extensive ongoing research study.

## Materials and methods

In this prospective, three-arm, parallel-group, randomized, active-controlled, open-label study, patients with MDD are being evaluated for the antidepressant effects of three drugs, namely vilazodone, escitalopram, and vortioxetine. Recruitment activities commenced in July 2022 at the Department of Psychiatry, Veer Surendra Sai Institute of Medical Sciences and Research (VIMSAR), Burla, Odisha, India. Before including their names in this research project, each participant or their relatives submitted written informed consent. Before study initiation, the Institutional Ethics Committee, VIMSAR, granted ethical approval (approval number: 029-2022/I-S-T/03 dated May 17, 2022). The study was prospectively registered with the Clinical Trial Registry, India (CTRI/2022/07/043808). The study was carried out in conformity with institutional policies, the Declaration of Helsinki, and the criteria for Good Clinical Practice set forth by the International Council for Harmonization.

Study participants

Males and females between the ages of 18 and 65 years diagnosed with MDD at the psychiatry outpatient department, VIMSAR, Burla, India, with a score of ≥ 24 on the HDRS-17 items version [22], were included. Patients with any psychotic symptoms or organic brain disease, those with a history of known side effects to study drugs, those with renal impairment (estimated glomerular filtration rate 45 mL/min/1.73m^2^), those who had cardiovascular events within the previous six months, those whose alanine transaminase (ALT) or aspartate transaminase (AST) levels were > 150% the upper limit of normal, those with serum triglyceride level > 400 mg/dL, and pregnant women and lactating mothers were excluded from this study. The investigators gave participants the power to revoke their consent at any time during the study without mentioning their reason.

Study design and endpoints

The qualified participants got assigned in a 1:1:1 ratio to be treated with either vilazodone tablets (20-40 mg once daily; group A), escitalopram tablets (10-20 mg once daily; group B), or vortioxetine tablets (5-20 mg once daily; group C). We accomplished this with blocks of sizes 12 and 24 through permuted block randomization. Plus, the randomization was stratified by gender (female or male) and the condition in question (treatment naïve or on antidepressant medications for < 6 months).

The change in HDRS score from baseline at week 12 served as the primary objective for this interim analysis. The change in MADRS score from baseline at week 12 and the incidence of adverse events in the study population served as the secondary endpoints. The per-protocol (PP) population functioned as the subject for the efficacy assessments, whereas the intent-to-treat (ITT) population worked as the subject for the safety assessments.

Study procedure

Throughout this study, all recruited patients received either tablet of vilazodone (20-40 mg, once daily orally; group A), a tablet of escitalopram (10-20 mg, once daily orally; group B), or a tablet of vortioxetine (5-20 mg, once daily orally; group C). The principal investigator provided these medicines to participants without any charge. The psychiatrist titrated the dose based on the participant's response to the prescribed medication. There was never any cross-over of study drugs. The pre-designed case record form was used to record the sociodemographic and clinical data pertinent to the participants. Each patient underwent a thorough physical and mental state examination at the baseline visit.

We scheduled follow-up appointments for the participants at four, eight, and 12 weeks following the first dose of their antidepressant treatment. All efficacy and safety parameters were assessed during each visit. The tools employed for the efficacy assessments were the HDRS-17 items version [22] and MADRS [23]. Safety analysis, i.e., incidence and seriousness of adverse events of the study population, were also evaluated at each visit. The study participants with either treatment failure or serious adverse events received rescue therapy with conventional antidepressant medication. Treatment failure was defined as the occurrence of any one of the following events: an increase in HDRS score of at least 3 points over the previous visit, an increase in MADRS score of at least 5 points over the last visit, and administration of any rescue medication in the investigator's best medical judgment.

Statistical analysis

The sample size was computed for the primary endpoint of the overall study. With a standard deviation (SD) of 2.0 and a mean difference of 10.0 in HDRS from baseline, it would necessitate the participation of 87 patients (29 in each group) to detect a change in HDRS with an 80% power at a 0.05 two-sided significance level. An aggregate of 96 patients (32 in each group), encompassing a 10% dropout or loss to follow-up, were slated for participating in the study. After the first 48 participants' 12-week visits culminated, we envisioned performing an interim analysis.

The data was verified for normality and homogeneity of variance using the Shapiro-Wilk test before performing any statistical analysis. The summary statistics for continuous and categorical variables were mean with SD or median with interquartile range (IQR) and frequency with proportion, respectively. Nonparametric analyses were carried out as needed. The three study groups' sociodemographic characteristics were compared using Fisher's exact test or Pearson's chi-square test. The Kruskal-Wallis test was employed to compare the HDRS and MADRS median scores. The Bonferroni test was deployed in the post-hoc analysis. For data analysis, we used R software (version 4.2.2) [24]. All statistical tests were two-tailed, and a p-value < 0.05 was deemed statistically significant.

## Results

**Figure 1:**
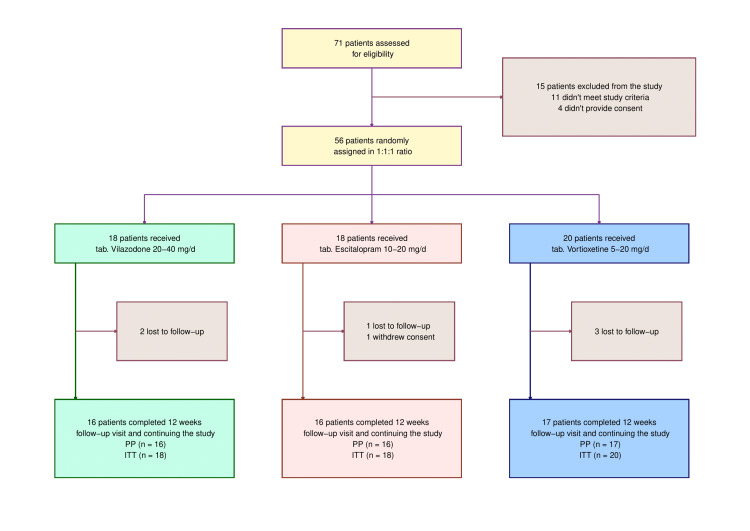
CONSORT diagram mg/d: milligram per day; PP: Per-protocol analysis; ITT: Intent-to-treat analysis; CONSORT: Consolidated Standards Of Reporting Trials

Eleven of the 71 patients subjected to the eligibility screening and four who declined consent were dropped from the study. The remaining 56 patients were subsequently randomized into one of three study groups. Six were lost to follow-up (two from the vilazodone arm, one from the escitalopram arm, and three from the vortioxetine arm), and one from the escitalopram arm revoked her consent. Forty-nine subjects were analyzed for efficacy assessments in this interim analysis (24 females and 25 males; 16 in the vilazodone arm, 16 in the escitalopram arm, and 17 in the vortioxetine arm). Fifty-six subjects made up the ITT population, who were analyzed for safety assessments (Figure 1). Participants in all three groups shared similar baseline traits (Table 1).

**Table 1:** Baseline characteristics of the per-protocol population (n = 49) The continuous variables were expressed as either mean ± standard deviation or median (inter-quartile range). The categorical values were presented as n (%). BMI: Body mass index; HDRS: Hamilton Depression Rating Scale-17 items version; MADRS: Montgomery Åsberg Depression Rating Scale.

	Total (n = 49)	Group A Vilazodone (n = 16)	Group B Escitalopram (n = 16)	Group C Vortioxetine (n = 17)	p-Value
Age (years)	43.9 ± 12.2	45.8 ± 11.2	41.4 ± 11.9	44.4 ± 13.7	0.618
Age group					
≤ 50 years	32 (65.3%)	11 (68.8%)	11 (68.8%)	10 (58.8%)	0.789
>50 years	17 (34.7%)	5 (31.2%)	5 (31.2%)	7 (41.2%)	
Gender					
Female	25 (51.0%)	8 (50.0%)	8 (50.0%)	9 (52.9%)	0.981
Male	24 (49.0%)	8 (50.0%)	8 (50.0%)	8 (47.1%)	
Duration of disease					
T/t naïve	25 (51.0%)	8 (50.0%)	8 (50.0%)	9 (52.9%)	0.981
< 6 months	24 (49.0%)	8 (50.0%)	8 (50.0%)	8 (47.1%)	
BMI (kg/m^2^)	27.3 ± 4.8	27.4 ± 4.1	27.6 ± 5.2	27.1 ± 3.9	0.128
HDRS	30.00 (29.00 - 31.00)	30.00 (29.00 - 31.00)	29.50 (29.00 - 30.00)	29.00 (29.00 - 32.00)	0.763
MADRS	36.00 (35.00 - 37.00)	36.00 (35.00 - 37.00)	36.00 (35.00 - 36.25)	36.00 (35.00 - 38.00)	0.789

**Figure 2:**
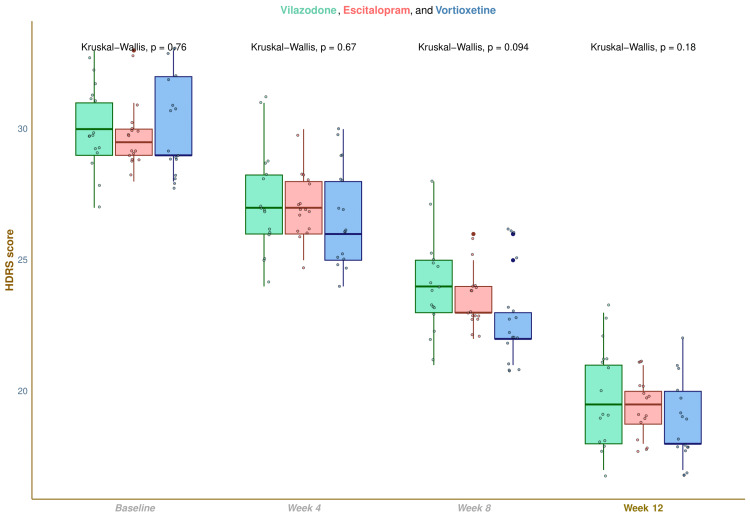
HDRS scores of participants at various time points The box-whisker and jitter plots show the HDRS scores of participants of all three groups. The inter-group comparisons at each visit were performed with the Kruskal-Wallis test. HDRS: Hamilton Depression Rating Scale-17 items version

**Figure 3:**
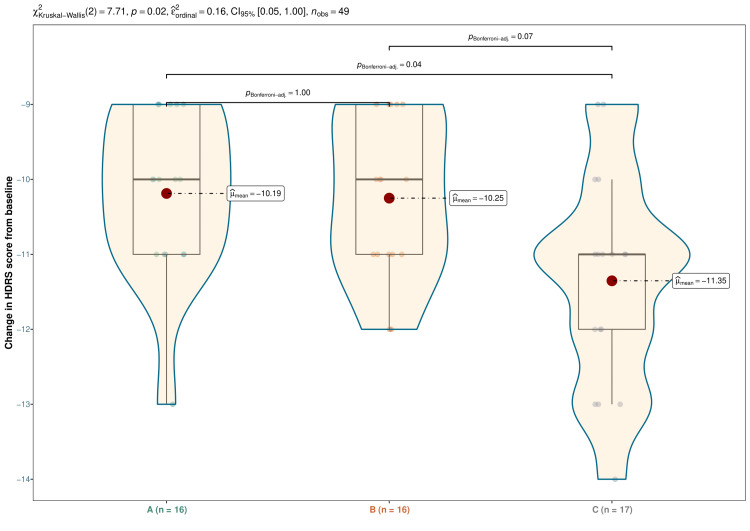
Post-hoc analysis of the difference in HDRS scores from baseline The box-whisker, violin, and jitter plots show the difference from baseline in HDRS scores of participants of all three groups. The dots indicate the mean changes. The inter-group comparison was performed with the Kruskal-Wallis test, followed by the Bonferroni test. HDRS: Hamilton Depression Rating Scale-17 items version

The median HDRS scores were 30.0 (29.0-31.0) at baseline, 27.0 (26.0-28.2) at four weeks, 24.0 (23.0-25.0) at eight weeks, and 19.5 (18.0-21.0) at 12 weeks in vilazodone group (difference from baseline: -10.0 (-11.0 to -9.0); p < 0.001), 29.5 (29.0-30.0) at baseline, 27.0 (26.0-28.0) at four weeks, 23.0 (23.0-24.0) at eight weeks, and 19.5 (18.8-20.0) at 12 weeks in escitalopram group (difference from baseline: -10.0 (-11.0 to -9.0); p < 0.001), and 29.0 (29.0-32.0) at baseline, 26.0 (25.0-28.0) at four weeks, 22.0 (22.0-23.0) at eight weeks, and 18.0 (18.0-20.0) at 12 weeks in vortioxetine group (difference from baseline: -11.0 (-12.0 to -11.0); p < 0.001), respectively (Figure 2). Following a 12-week intervention, an intragroup comparison illustrated that the HDRS scores for all three groups had substantially dwindled (p < 0.001). Additionally, intergroup comparisons revealed statistically significant differences (p = 0.021). We adopted the Bonferroni correction while performing the post-hoc analysis on the same data. It emphasized that the differences between the groups receiving vortioxetine and vilazodone were statistically significant (p = 0.04) (Figure 3).

**Figure 4:**
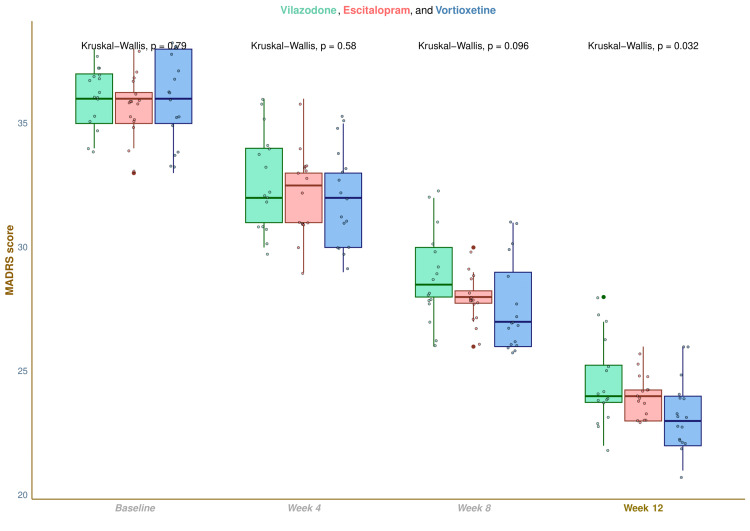
MADRS scores of participants at various time points The box-whisker and jitter plots show the MADRS scores of participants of all three groups. The inter-group comparisons at each visit were performed with the Kruskal-Wallis test. MADRS: Montgomery Åsberg Depression Rating Scale

**Figure 5:**
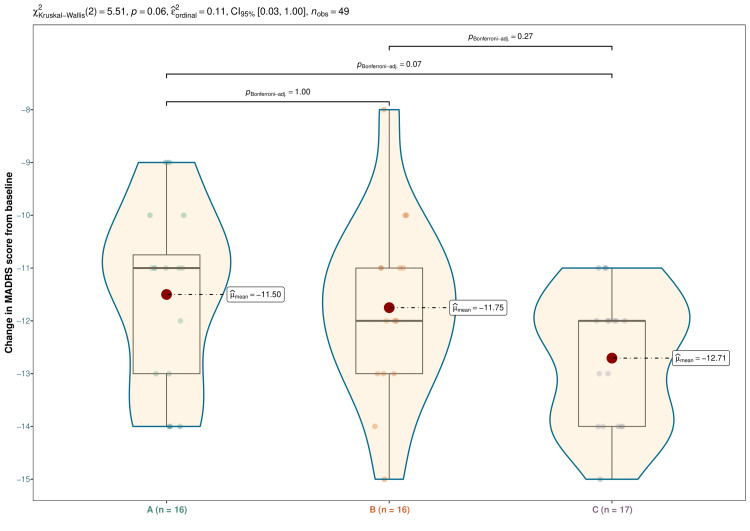
Post-hoc analysis of the difference in MADRS scores from baseline The box-whisker, violin, and jitter plots show the difference from baseline in MADRS scores of participants of all three groups. The dots indicate the mean changes. The inter-group comparison was performed with the Kruskal-Wallis test, followed by the Bonferroni test. MADRS: Montgomery Åsberg Depression Rating Scale

The median MADRS scores were 36.0 (35.0-37.0) at baseline, 32.0 (31.0-34.0) at four weeks, 28.5 (28.0-30.0) at eight weeks, and 24.0 (23.8-25.3) at 12 weeks in vilazodone group (difference from baseline: -11.0 (-13.0 to -10.8); p < 0.001), 36.0 (35.0-36.3) at baseline, 32.5 (31.0-33.0) at four weeks, 28.0 (27.8-28.3) at eight weeks, and 24.0 (23.0-24.3) at 12 weeks in escitalopram group (difference from baseline: -12.0 (-13.0 to -11.0); p < 0.001), and 36.0 (35.0-38.0) at baseline, 32.0 (30.0-33.0) at four weeks, 27.0 (26.0-29.0) at eight weeks, and 23.0 (22.0-24.0) at 12 weeks in vortioxetine group (difference from baseline: -12.0 (-14.0 to -12.0); p < 0.001), respectively (Figure 4). After 12 weeks of intervention, an intragroup comparison disclosed that all three drugs substantially lowered MADRS scores (p < 0.001). Nevertheless, the intergroup comparison yielded non-significant differences (p = 0.064). We applied the Bonferroni correction to perform the same post-hoc analysis. It claimed no statistically significant differences in MADRS scores across the three study groups (Figure 5).

The adverse events faced by each participant were assessed using an intent-to-treat (ITT) analysis. There were 31 adverse events altogether. The participants on escitalopram (14) endured the majority of events, closely followed by those receiving vilazodone (10) and vortioxetine (7). None of the events, according to National Cancer Institute-Common Terminology Criteria for Adverse Events (NCI-CTCAE) version 5.0, seemed serious. Most events (26) were mild, keeping with Hartwig's severity scale. Four events fell into the moderate category, whereas one was severe. In the wake of adverse events, two participants left the study. Their discontinuance was brought on by diarrhea (one each from vilazodone and escitalopram groups). The most frequent adverse event encountered by the study population was nausea and vomiting (10), followed by disturbances in the sleep duration and architecture (7), dizziness (5), diarrhea (5), rash (3), headache (2), and hypotension (2) (Table 2).

**Table 2:** Adverse events in the intent-to-treat population (n = 56) The seriousness and severity of the adverse events were evaluated with NCI-CTCAE version 5.0 and Hartwig’s severity scale, respectively. The p-values were calculated using the chi-square (χ2) test or the Fisher's exact test NCI-CTCAE: National Cancer Institute Common Terminology Criteria for Adverse Events; NA: Not applicable

	Group A Vilazodone (n=18)	Group B Escitalopram (n=18)	Group C Vortioxetine (n=20)	p-Value
Total adverse events	14	10	7	0.027
Serious (> grade 3)	0	0	0	NA
Severity of events				0.015
Mild	11	9	6	
Moderate	2	1	1	
Severe	1	0	0	
Events led to discontinuation	1	1	0	0.731
Individual events				
Nausea and vomiting	5	3	2	0.043
Sleep disturbances	4	2	1	0.039
Dizziness	2	2	1	0.094
Diarrhea	3	1	1	0.047
Skin rash	1	1	1	0.868
Headache	1	1	0	0.619
Hypotension	1	0	1	0.553

## Discussion

According to this interim analysis of a continuing study, vortioxetine, which modulates serotonin activity, led to a considerable diminution in both HDRS and MADRS scores at 12 weeks compared to escitalopram, which impedes serotonin reuptake, and vilazodone, which provides additional 5-HT1A receptor partial agonistic action to serotonin reuptake inhibition. Arguably, the bulk of the side effects were minor and non-serious. The vortioxetine group possessed a more favorable safety profile and patient tolerability. These results concur with those from two recent meta-analyses [20,21].

The dosages for those participating in the two intervention arms were 20-40 mg of vilazodone and 5-20 mg of vortioxetine per day, respectively, whereas the dosage for those enrolled in the control arm was 10-20 mg per day of escitalopram. Escitalopram has a single mechanism of action as it is an SSRI, while vilazodone additionally carries the plus of being a partial agonist at the 5-HT1A receptor. Vortioxetine, however, interferes with serotonin transport and directly modulates serotonin receptors. In light of these extra upsides, the corresponding reductions in HDRS and MADRS scores in the vortioxetine group were more pronounced than in the other two study groups. These findings, demonstrating some contrast, propose vortioxetine monotherapy as a potential candidate in the treatment of MDD.

Throughout the study, all participants got free medications regardless of the groups to which they were assigned. This might have fostered a high attrition rate, which in turn possibly aided the antidepressant effects of the drugs, as evidenced by the plummeted scores and the gradual easing of depression symptoms over time. Vortioxetine is anticipated to enhance the treatment of MDD as a first-line antidepressant in the years to come relying on the declines in HDRS and MADRS scores and the nature of adverse events observed in the vortioxetine group [16,19]. Effective antidepressant action, according to a recent systematic review and network meta-analysis [20], calls for a high attrition rate, more frequent periodic visits with real-time interaction, multi-tool examination of depressed symptoms, and a lower incidence of adverse reactions. This study addresses the entirety of these elements, which explains the outcomes to some extent. However, these findings should be viewed as speculative since this was only an interim assessment of a more extensive research project. Further assessments are required to check whether the effect size and safety profile are maintained after a longer follow-up.

This study's key strengths were implementing permuted block randomization and assessment of MDD with two widely-accepted tools, i.e., HDRS [22] and MADRS [23]. The added advantages were the higher follow-up visits and analysis of both the PP and ITT populations. There are certain limitations to our study as well. First, dropouts and possible reporting biases in the context of adverse events could be attributed to the open-label study design. It impacted the study's internal validity. Second, no fee was received for the antidepressants used in this study. The study drugs' affordability might hamper the generalizability of the study's findings. Third, these findings depend on an interim analysis of a more extensive, advancing study. There needs to be more certainty regarding the accuracy between these data and those from the more comprehensive study. Fourth, there are numerous aspects and contributing factors to depression. In a real-world setting, it is challenging to establish if long-term antidepressant therapy is effective.

## Conclusions

After 12 weeks of intervention, vortioxetine significantly lowered HDRS and MADRS scores as weighed against vilazodone and escitalopram, according to this continuing study's interim analysis. Additionally, it had a safer profile than the other two medications. The long-term antidepressant effects of these drugs have to be evaluated further.
